# Phytoecdysteroids as modulators of the *Toxoplasma gondii* growth rate in human and mouse cells

**DOI:** 10.1186/s13071-015-1019-7

**Published:** 2015-08-15

**Authors:** Katarzyna Dzitko, Marcin Mikołaj Grzybowski, Jakub Pawełczyk, Bożena Dziadek, Justyna Gatkowska, Paweł Stączek, Henryka Długońska

**Affiliations:** Department of Immunoparasitology, Faculty of Biology and Environmental Protection, University of Łódź, Banacha 12/16, 90-237 Łódź, Poland; Institute of Medical Biology of the Polish Academy of Sciences, Lodowa 106, 93-232 Łódź, Poland; Department of Microbial Genetics, Faculty of Biology and Environmental Protection, University of Łódź, Banacha 12/16, 90-237 Łódź, Poland

## Abstract

**Background:**

Searching for new effective drugs against human and animal toxoplasmosis we decided to test the anti-*Toxoplasma* potential of phytoecdysteroids (α-ecdysone and 20-hydroxyecdysone) characterized by the pleiotropic activity on mammalian organisms including the enhancement of host’s anti-parasitic defence. This objective was accomplished by the *in vitro* evaluation of *T. gondii* growth in phytoecdysteroid-treated immunocompetent cells of selected hosts: humans and two strains of inbred mice with genetically determined different susceptibility to toxoplasmosis.

**Methods:**

Peripheral mononuclear blood cells were isolated from *Toxoplasma*-positive and *Toxoplasma*-negative women (*N* = 43) and men (*N* = 21). Non-infected mice (C57BL/6, *N* = 10 and BALB/c, *N* = 14) and mice (BALB/c, *N* = 10) challenged intraperitoneally with 5 tissue cysts of the *T. gondii* DX strain were also used in this study as a source of splenocytes. The effects of phytoecdysteroids on the viability of human PBMC and mouse splenocytes were evaluated using the MTT assay. The influence of phytoecdysteroids on PBMCs, splenocytes and *T. gondii* proliferation was measured using radioactivity tests (the level of 3[H] uracil incorporation by toxoplasms or 3[H] thymidine by PBMCs and splenocytes), which was confirmed by quantitative Real-Time PCR. Statistical analysis was performed using SigmaStat 3.5 (Systat Software GmbH). The best-fit IC50 curves were plotted using GraphPad Prism 6.0 (GraphPad Software, Inc.).

**Results:**

Our results showed that phytoecdysteroids promote the multiplication of *Toxoplasma* in cultures of human or murine immune cells, in contrast to another apicomplexan parasite, *Babesia gibsoni*. Additionally, the tested phytoecdysteroids did not stimulate the *in vitro* secretion of the essential protective cytokines (IFN-γ, IL-2 and IL-10), neither by human nor by murine immune cells involved in an effective intracellular killing of the parasite.

**Conclusions:**

Judging by the effect of phytoecdysteroids on the *T. gondii* proliferation, demonstrated for the first time in this study, it seems that these compounds should not be taken into consideration as potential medications to treat toxoplasmosis. Phytoecdysteroids included in the food are most likely not harmful for human or animal health but certain nutrients containing ecdysteroids at high concentrations could promote *T. gondii* proliferation in chronically infected and immunocompromised individuals. In order to assess the real impact of ecdysteroids on the course of natural *T. gondii* invasion, *in vivo* research should be undertaken because it cannot be ruled out that the *in vivo* effect will be different than the *in vitro* one. However, taking into account the possible stimulating effect of ecdysteroids on some opportunistic parasites (such as *Toxoplasma* or *Strongyloides*) further studies are necessary and should focus on the mechanisms of their action, which directly or indirectly enhance the parasite growth. Since ecdysteroids are considered as potential drugs, it is essential to determine their effect on various parasitic pathogens, which may infect the host at the same time, especially in immunocompromised individuals.

## Background

*Toxoplasma gondii* is a cosmopolitan intracellular protozoan that causes toxoplasmosis in birds, mammals and humans. Statistical data concerning the prevalence of toxoplasmosis indicated that at least one-third of human population has had contact with this parasite. The high prevalence of *T. gondii* infection (e.g., in some parts of Europe 58–90 %) is related to sex, age, culinary habits, personal hygiene, condition of the immune system and dysfunctions of the endocrinal network [1–3]. Despite the significant progress in the research on the biology of *T. gondii* invasion, the proteome of this protozoan and the immunity it induces, no preventive vaccines for humans have been developed yet.

*T. gondii* infection leads to congenital or acquired postnatal toxoplasmosis characterized by diverse forms and symptoms. In immunocompetent humans, the infection is usually asymptomatic as the primary immunological response quickly limits the parasite replication and its spreading. Thus, symptomatic toxoplasmosis occurs infrequently. Quickly replicating tachyzoites are converted into bradyzoites and become enclosed in tissue cysts, which are a sign of chronic toxoplasmosis [4]. Recent studies demonstrated that a long-term presence of the parasite is not neutral to the host. It is increasingly often postulated that there is a causal connection between the *T. gondii* carriage and the increased risk of neurologic diseases, such as schizophrenia [5], Parkinson’s disease [6] or epilepsy [7]. Moreover, a very significant clinical problem is posed by congenital toxoplasmosis, resulting from the primary infection of a mother during pregnancy and transmission of the parasite to a fetus with an immature immune system. In chronically infected women there is usually no placental parasite transmission to a fetus, thus, an especially interesting group for clinicians and immunoparasitologists are seronegative women at a child-bearing age, in whom the development of a primary infection may cause a miscarriage or congenital defects of the offspring, such as hydrocephaly or microcephaly, chorioretinitis and intracranial calcification, which constitute the so-called “Sabin-Feldman triad”. This parasite may also cause serious medical disorders in humans related to the unchecked proliferation of the protozoan in immunocompromised patients. A weakened immune system is not able to inhibit the parasite replication during the reactivation of a latent infection or the reinvasion by a new strain of a different genotype. Uncontrolled *T. gondii* invasion in such cases can become a direct cause of death [8, 9].

In the treatment of most clinical forms of toxoplasmosis, the antagonists of folic acid are used: pyrimethamine in combination with sulfadiazine [10]. Although such therapy is often effective, it may cause multiple side effects, such as bone marrow suppression, which leads to the necessity of concurrent administration of folic acid [11]. Additionally, because of the frequent intolerance to sulfadiazine, it is often necessary to administer other medications, e.g., clindamycin or co-trimoxazole. Numerous drugs used in the treatment of toxoplasmosis cause many side effects including allergic reactions, leukopenia, anemia, thrombocytopenia, cardiac dysrhythmia, as well as digestive tract disorders and skin pigmentation disorders. Hence, searching for less toxic drugs that will effectively act against all life-stages of the parasite is crucial for the improvement of *T. gondii* infections treatment.

As current chemotherapy is still not satisfactory, many attempts are made at developing specific immunoprophylaxis (a vaccine that will induce strong protective immunity against toxoplasmosis) [12–14] and obtaining not only new drugs [15–18] but also alternative therapeutical preparations derived from plants [19, 20]. Such alternative methods motivate scientists to search for new strategies to fight toxoplasmosis [3, 21]. Because of the pleiotropic activity of phytoecdysteroids on mammalian organisms (including humans), they gain interest as agents that potentially enhance the antibacterial, antifungal and antiparasitic activity of host cells. Such studies are still scarce, however, the obtained results are very promising and give hope for future application of ecdysteroids in the chemotherapy of parasitoses. It has been shown that certain plant extracts are able to suppress the growth of some *Apicomplexa* protozoans. For instance, the infusion of *Artemisia annua*, rich in artemisinin and its derivatives, inhibited the proliferation of *T. gondii* and *Plasmodium falciparum* [22]. Interestingly, the extracts of *Sophora flavescens* and *Zingiber officinale* proved to be highly selective against the RH strain of *T. gondii* [23]. These exemplary results indicate that plants might be a valuable source of drugs against parasitic diseases, including toxoplasmosis. Novel plant-derived compounds might be used to replace or complement currently used chemotherapeutic agents. Furthermore, 20-hydroxyecdysone obtained from the plant *Arcangelisia flava* demonstrated an inhibitory effect on the protozoan parasite *Babesia gibsoni* [24], the etiological agent of babesiosis in dogs, which, like *T. gondii*, belongs to the *Apicomplexa* phylum. It raises a question whether 20-hydroxyecdysone might have a similar effect on *T. gondii*.

## Methods

### Ethical approval

All experimental procedures on female (10–12 weeks old) inbred C57BL/6 (H-2^b^) and BALB/c (H-2^d^) mice kept under standard laboratory conditions, were conducted according to the guidelines of the Local Ethics Commission for Experiments on Animals in Lodz (licence number 39/ŁB475/2009). Mice were used for the propagation of *Toxoplasma* tachyzoites and to obtain splenocytes as host cells for *in vitro* parasite proliferation tests.

### DX and RH *Toxoplasma gondii* cultivation

*T. gondii* tissue cysts of the low virulence DX strain (intraspecies type II) were used for mice challenge. DX strain was maintained in C57BL/6 mice by the passage of five brain cysts administered intraperitoneally every 2 months.

The tachyzoites of the *T. gondii* RH strain – intraspecies type I (ATCC® Number 50174™) were maintained through passages on female C57BL/6 and BALB/c mice with genetically determined high and low susceptibility (respectively) to *T. gondii* infection. The tachyzoites washed out from the peritoneal cavity were expanded *in vitro* on the L929 cell line (ATTC® Catalog No. CCL-1, mouse fibroblasts) for one infection cycle. Co-cultures of tachyzoites and host cells were cultivated in Iscove’s Modified Dulbecco’s Medium (IMDM), supplemented with 2 mM L-glutamine, 100 U/mL penicillin, 100 μg/mL streptomycin, 5 × 10^−5^ M β-mercaptoethanol and 5 % Fetal Bovine Serum (FBS). All commercial reagents were purchased from Sigma-Aldrich (Poland). Extracellular parasites were pelleted from the culture supernatant of the host cells, passed through a 5 μm pore filter (Sartorius, Poland) and centrifuged (720 × *g*, 15 min, 24 °C).

### Blood collection, processing and human peripheral blood mononuclear cells isolation

Clinical and serological analyses were performed on a group of 43 women and 21 men in the age range of 20–67 years (mean value 31.6 years; median 28). Only those patients who voluntarily signed a written agreement were involved in the project. The study was approved by the Local Committee of Ethics in Science (licence number 11 ⁄ 10 ⁄ 2005) and conducted in accordance with the Helsinki Declaration.

Clinical specimens for the study were collected from each patient and consisted of serum samples (used to determine the presence of the anti-*Toxoplasma* IgG antibody) and peripheral blood samples stabilized with EDTA (as a source of peripheral blood mononuclear cells – PBMCs). PBMCs were isolated from the blood samples using a Leucosept-Tube with Ficoll-Paque Plus® (Greiner Bio-One, Germany) according to the manufacturer’s instructions. The final number of PBMCs was adjusted to 2.5 × 10^6^ cells ⁄mL and they were used as host cells for *T. gondii in vitro* proliferation tests.

### Mouse splenocytes and brains isolation

Non-infected C57BL/6 mice (*N* = 10), BALB/c mice (*N* = 14) and the ones challenged intraperitoneally with 5 tissue cysts of the *T. gondii* DX strain (non-lethal dose) BALB/c mice (*N* = 10) were also used in this study. Four weeks after the challenge and directly prior to the experiment the animals were euthanized and the spleens and brains were isolated.

The brain of each mouse was homogenized by 10 passages through each of 19-, 20- and 21-gauge needles and finally suspended in 2 mL of PBS. The mean brain cyst load was determined by counting 25 μL samples of the homogenate under an Olympus inverted optical microscope at 200 × magnification. All samples were counted in duplicate. Challenged mice marked as *Toxoplasma*-positive and non-infected mice marked as *Toxoplasma*-negative were used for spleen isolation.

Single cell suspensions of splenocytes were obtained by spleen homogenization as described in [13]. The final spleen cells number was adjusted to 2.5 × 10^6^ cells ⁄mL and used as host cells for *T. gondii in vitro* proliferation tests.

### *In vitro* studies on human PBMC and mouse splenocytes

#### Cell viability assay

The effects of phytoecdysteroids on the viability of human PBMC and mouse splenocytes were evaluated using the MTT [3-(4,5-dimethylthiazol-2-yl)-2,5-diphenyltetrazolium bromide] assay. The MTT assay was used according to international standards: ISO 10993–5:2009(E), *Biological evaluation of medical devices*, Part 5: *Tests for in vitro cytotoxicity*. The cells were placed into 96-well plates (Falcon) at a density of 2.5 × 10^5^/100 μL/well in culture medium and 100 μL of tested phytoecdysteroids were added to the final concentrations of: 2; 10; 20 μg/mL of α-ecdysone (Sigma-Aldrich) or 2; 20; 100 μg/mL of 20-hydroxyecdysone (Sigma-Aldrich). Cells cultured in the non-supplemented medium or stimulated with concanavalin A (ConA) (Sigma-Aldrich) at a concentration of 2.5 μg/mL served as a negative or positive control of proliferation, respectively. Afterwards, the cells were exposed to the tested compounds for 24 h. Then, 1 mg/mL MTT (50 μL/well) was added to each well, incubated at 37 ° C, 10 % CO_2_ for 2 h and developed as described in [16]. The results were expressed as a viability percentage compared to the 2.5 % DMSO treated controls. All experiments were performed in triplicate.

#### Influence of phytoecdysteroids on PBMCs, splenocytes and *T. gondii* proliferation

The human PBMC and mouse splenocytes were seeded in triplicate at 2.5 × 10^5^ cells per well in 96-well tissue culture plates (Falcon) in: 100 μl of culture medium with an equal number (5 × 10^5^/50 μL/each well, ratio 1 : 2) of *T. gondii* tachyzoites or only in 150 μl of culture medium. Then 50 μL of culture medium with tested phytoecdysteroids was added to the final concentrations of: 2; 10; 20 μg/mL of α-ecdysone (Sigma-Aldrich) and 2; 20; 100 μg/mL of 20-hydroxyecdysone (Sigma-Aldrich). Cells cultured in the non-supplemented medium or stimulated with concanavalin A (ConA) (Sigma-Aldrich) at a concentration of 2.5 μg/mL served as a negative or positive control of proliferation, respectively. The cultures were incubated at 37 °C for 48 h in humidified atmosphere with 10 % CO_2_ and 1 μCi of [3H]uracil (Moravek Biochemicals Inc., USA) was then applied to each well for the last 20 h. After the microscopic examination, the plates were frozen at −20 °C and stored. Directly before the radioactivity measurement, the plate was thawed and tachyzoites and cells (PBMCs or splenocytes) were harvested with a semiautomatic cell harvester (SKATRON Instruments, Norway). Radioactivity (the level of 3[H] uracil incorporation by toxoplasms or 3[H] thymidyne by PBMCs and splenocytes) was measured using a 1450 Microbeta Plus Liquid Scintillation Counter (Wallac Oy, Finland). The cpms of control host cells (below 250/microculture) were subtracted from cpms of *T. gondii* infected microcultures.

#### Quantitative Real-Time PCR

*T. gondii* genomic DNA was isolated from the tachyzoites of the RH strain with the Wizard® SV Genomic DNA Purification System (Promega, Madison, WI, USA) according to the manufacturer’s instruction. The DNA concentration and purity were measured using a NanoPhotometer (Implen, München, Germany), while the integrity of the extracted DNA was tested using an ethidium bromide-stained agarose gel. DNA was used for the detection and quantitation of *T. gondii* in analyzed samples and it was stored at −20 °C. A quantitative real-time PCR (qRT-PCR) assay, targeting B1 gene [25] was performed to detect and to quantitate *T. gondii* in analyzed samples according to the modified protocol of Wahab et al*.* [26]. All samples were analyzed in triplicate.

#### Antibody and cytokine ELISAs

The *T. gondii* serological status of all human sera was checked by the determination of anti-*T. gondii* IgG and IgM antibodies using commercially available ELISA kits (NovaLisa™ IgG and NovaLisa™ IgM *μ*-capture; Nova-Tec Immundiagnostica GmbH, Germany). Sera were recognized as IgM-positive at NTK (NovaTec Units, NovaTec Immundiagnostica GmbH) level >11 and IgG-positive at >35 IU ⁄ mL. Overall, it was found that almost one-third of the tested volunteers were infected by *T. gondii* and the seroprevalence among men was slightly higher (38 %, *N* = 21) than among women (29 %, *N* = 43).

The levels of human and mice IFN-γ, IL-2, IL-4 and IL-10 were measured in the human PBMC and mouse splenocytes culture supernatants using commercially available ELISA sets (OptEIA™, BD Biosciences, Poland). The supernatants were collected at 24 h for IL-4, 48 h for IL-2 and 72 h for IFN-γ and IL-10 from non-infected cultures and after *Toxoplasma* infection. The assays were carried out according to the manufacturer’s instructions. The detection limits for each cytokine were as follows: 3.1 pg/mL (mouse IL-2), 4.7 pg/mL (human IFN-γ), 7.8 pg/mL (human IL-10, mouse IL-4) and 31.3 pg/mL (mouse IL-10 and IFN-γ).

#### Statistics

Statistical analysis was performed using SigmaStat 3.5 (Systat Software GmbH). The Kolmogorov-Smirnov test was applied for assessing the normality of the data distribution. The t-student or U-Mann–Whitney test was used for comparing two groups. For multiple comparisons the Kruskal-Wallis test or one-way ANOVA were performed. Differences with a two-tailed value of *p* < 0.05 were considered as statistically significant. The best-fit IC_50_ curves were plotted using GraphPad Prism 6.0 (GraphPad Software, Inc.).

## Results and Discussion

*T. gondii* has developed numerous mechanisms which enable it to colonize various cell types, alter their function and use them as a reservoir of infection. While *T. gondii* reproduces sexually only in feline enterocytes [27], it uses predominantly neural and muscle cells to survive and form tissue cysts [28]. Moreover, the parasite hijacks blood nucleated cells turning them into Trojan horses so that the infection disseminates throughout the host’s organism. With consideration to the variety of hosts and tissues which can be invaded by this parasite, two different cell types were chosen for the research: human peripheral blood mononuclear cells (PBMCs) and murine splenocytes. We also used two different mouse strains characterized by low (BALB/c) and high (C57BL/6) innate susceptibility to toxoplasmosis. Moreover, both human and mouse cells were collected from *T. gondii* seronegative as well as seropositive individuals.

In the first step of experiments aimed at checking the anti-toxoplasmic activity of selected phytoecdysteroids, we assessed the potential cytotoxicity of α-ecdysone and 20-hydroxyecdysone using the MTT assay and [^3^H]-thymidine incorporation test. As we expected [29, 30], all cells responded positively after stimulation with the concanavalin A mitogen (*p* < 0.05) in the control microcultures. Importantly, neither phytoecdysteroids showed cytotoxic effect on PBMCs (Table 1) nor murine splenocytes (data not shown).

**Table 1:** Metabolic activity of human PBMCs

		Metabolic activity [%] of human PBMCs			
		24 h	48 h	72 h	
Cmpd.	C [μg/mL]	MTT		MTT	[3H]-thymidine
ConA	2.5	91.62 ± 19.97	115.67 ± 24.71	^*^189.69 ± 42.55	^*^926.57 ± 169.21
α-ecdysone	2	69.76 ± 32.29	84.63 ± 25.67	76.04 ± 26.90	92.27 ± 18.25
	10	89.51 ± 22.83	93.81 ± 30.35	85.59 ± 24.40	94.19 ± 19.46
	20	91.97 ± 25.74	94.73 ± 23.05	97.07 ± 26.87	94.08 ± 26.97
20-hydroxy ecdysone	2	97.32 ± 20.23	97.69 ± 29.34	105.03 ± 22.52	99.21 ± 21.45
	20	100.92 ± 15.48	102.92 ± 27.78	107.60 ± 20.78	100.13 ± 27.84
	100	95.68 ± 15.57	102.05 ± 25.34	106.62 ± 22.18	92.71 ± 26.51
sulfadiazine	2	95.74 ± 12.74	103.15 ± 27.05	107.30 ± 26.87	91.24 ± 22.48
	20	98.37 ± 18.35	104.37 ± 20.69	107.00 ± 26.21	96.23 ± 24.57
	100	100.63 ± 19.82	106.38 ± 26.83	104.51 ± 21.83	89.68 ± 28.18

PBMCs isolated from individuals (*N* = 61) were tested in the presence of phytoecdysteroids and sulfadiazine in the concentration range between 2 and 100 μg/mL ± SD at the incubation time from 24 to 72 h. The statistically significant differences (*p* < 0.05) were labeled with an asterisk (*); to calculate the decrease in the metabolic rate compared to the untreated blank, the following equation was used: viability measured using the MTT assay [%] = 100 × sample OD_570_ (the mean value of the measured optical density of the test samples) / blank OD_570_ (the mean value of the measured optical density of the untreated cells); using the [^3^H]-thymidine test: 100 × mean cpm incorporated into treated cells / mean cpm incorporated into cells cultured without phytoecdysteroids

The effect of two selected phytoecdysteroids (α-ecdysone and 20-hydroxyecdysone) on *T. gondii* proliferation was assessed by the [^3^H]-uracil radioisotope method, in which the tritiated uracil is selectively incorporated into the parasite’s nucleic acids by a highly active uracil phosphoribosyltransferase and uridine phosphorylase [31]. The qRT-PCR test was additionally performed to confirm the observation. The obtained results (Table 2) showed that neither compound proved to have inhibitory effect on *T. gondii* growth in human leukocyte cultures. Moreover they even stimulated the tachyzoites to proliferate. Furthermore, we observed the same phenomenon regardless of the serological status of the host cell donors. *T. gondii* proliferated more intensively as the concentration of α-ecdysone increased and at 20 μg/mL the number of the parasite’s daughter cells in PBMCs was higher by 15.05 ± 21.26 % (*p* < 0.05) for seronegative and by 40.74 ± 57.33 % (*p* < 0.05) for seropositive individuals, compared to controls. In the case of 20-hydroxyecdysone, a significant increase in the parasite proliferation was observed only at the concentration of 20 μg/mL, i.e., 17.23 ± 42.05 % (*p* < 0.05) for seronegative and 11.68 ± 18.84 % (*p* < 0.05) for seropositive individuals. As shown in Table 2, these results were confirmed by qRT-PCR.

**Table 2:** Effect of phytoecdysteroids on the intensity of *T. gondii* proliferation in human PBMCs

Cmpd.	C [μg/mL]	Proliferation of parasites [%] in human PBMCs (*N* = 61)			
		*Toxo*-seronegative (*N* = 40)		*Toxo*-seropositive (*N* = 21)	
		[^3^H] uracil	qRT-PCR	[^3^H] uracil	qRT-PCR
α-ecdysone	2	90.59 ± 19.20	nt	86.04 ± 27.57	nt
	10	105.35 ± 25.33	nt	98.06 ± 21.55	nt
	20	^*^115.09 ± 21.26	^*^120.73 ± 27.06	^*^140.74 ± 57.33	^*^138.99 ± 51.02
20-hydroxy ecdysone	2	101.93 ± 23.22	nt	^*^107.98 ± 22.54	nt
	20	^*^117.23 ± 42.05	^*^122.82 ± 30.58	^*^111.68 ± 38.84	^*^109.32 ± 26.28
	100	105.50 ± 32.48	nt	95.77 ± 21.85	nt
ConA	2.5	92.62 ± 29.98	nt	102.62 ± 25.35	nt

PBMCs isolated from individuals (*N* = 61) were tested in the presence of phytoecdysteroids and ConA in the concentration range between 2 and 100 μg/mL ± SD. Incubation period was 72 h. The statistically significant differences (*p* < 0.05) were labeled with an asterisk (*). To calculate the intensity of *Toxoplasma* proliferation compared to the untreated blank, the following equation was used: proliferation [%] = 100 × mean cpm incorporated into the treated cells in the [^3^H] uracil test or B1 gene in the qRT-PCR assay / mean cpm incorporated into the cultured cells or B1 gene without phytoecdysteroids

**Fig. 1:**
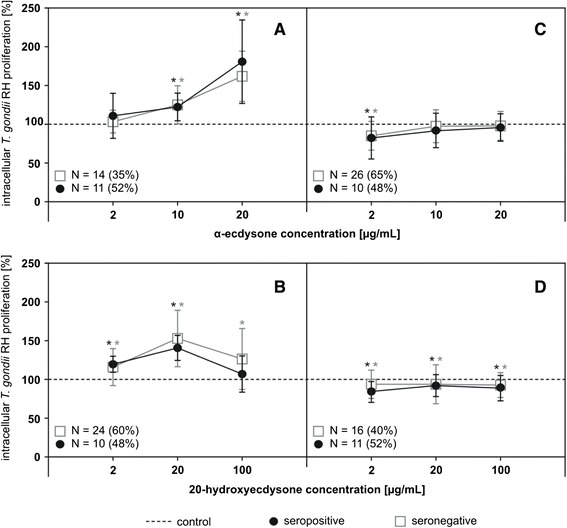
Effect of α-ecdysone and 20-hydroxyecdysone on *T. gondii* proliferation in PBMCs. PBMCs from *Toxo*-seronegative (□) and *Toxo*-seropositive (●) individuals, in the groups with observed, statistically significant (A, *N* = 26 and B, *N* = 34) and non-observed (C, *N* = 36 and D, *N* = 26) *T. gondii* proliferation, in the presence of the tested compounds, after 72 h of incubation

Due to a relatively high spread of the obtained results, we performed a further analysis of the detailed data (Fig. 1). The analysis led to the conclusion that within the seronegative and seropositive individuals, two subgroups could be identified: individuals whose cells were influenced by the tested compounds (Fig. 1, group A and B) or those who did not seem to be affected (Fig. 1, group C and D). In the groups A and B the level of parasite proliferation in PBMCs, in the presence of the highest tested concentration of α-ecdysone increased by 62 % ± 32 (*p* < 0.05) or 81 % ± 53 (*p* < 0.05) compared to the average values in seronegative and seropositive groups, respectively. At 20 μg/mL of 20-hydroxyecdysone a similar increase was observed, i.e., 52 % ± 36 (*p* < 0.05) in seronegative and 40 % ± 17 (*p* < 0.05) in seropositive individuals, as compared to controls.

**Fig. 2:**
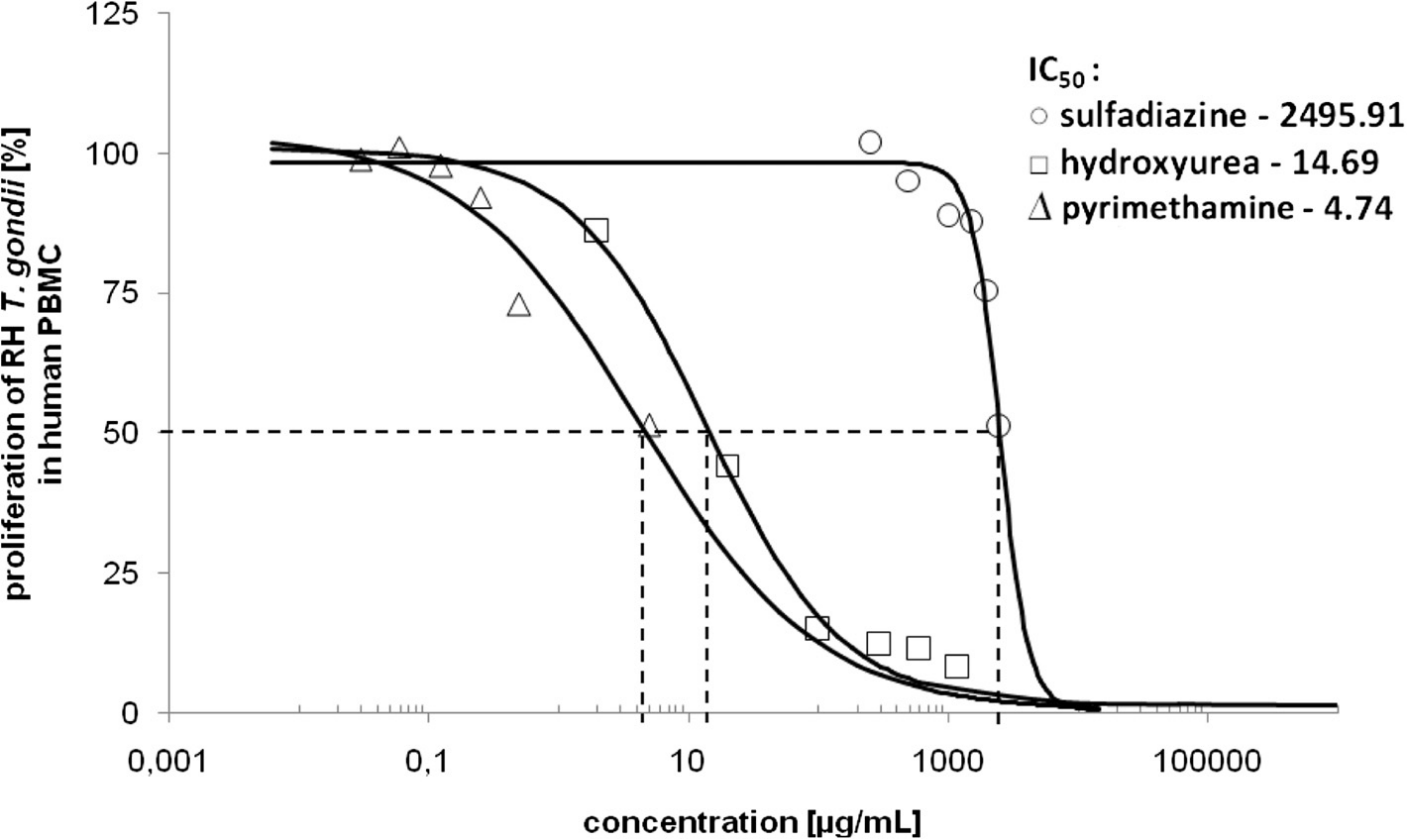
Estimation of IC_50_ [μg/mL] values of sulfadiazine, pyrimethamine and hydroxyurea in *T. gondii* infected human PBMCs

It was also found that the addition of concanavalin A to the tested cultures did not affect the final level of *T. gondii* proliferation; most likely because the host cells were unable to respond to a mitogen as quickly as the invasion of the parasites occurred. For comparative purposes we tested parallelly three different chemical agents able to restrict RH *T. gondii* growth: sulfadiazine and pirymethamine (used as recommended drugs in the therapy of toxoplasmosis) as well as hydroxyurea (used in anti-tumor therapy or in research) as a positive controls. In Fig. 2 we present the IC_50_ values for sulfadiazine (IC_50_ = 2495.91 μg/mL), pyrimethamine (IC_50_ = 14.69 μg/mL) and hydroxyurea (IC_50_ = 4.74 μg/mL) against RH *T. gondii* proliferating in normal human cells (PBMCs) at the parasite-host cell ratio of 2:1. Numerous studies proved that *T. gondii* susceptibility to a drug depends both on the invading strain and the host cells to be infected. Using the RH strain and different normal host cells, i.e., human MRC-5 [32], Vero [33], HFF [34] the IC_50_ values of sulfadiazine ranged from 2.5 to 77 μg/mL. In contrast to this, the use of human carcinoma HEp-2 [35] or HeLa [36] cells changed the IC_50_ to, 600–700 or >1000 μg/mL, respectively. As our observations reveal, the determination of the IC_50_ for a certain parasite strain is closely related to the type of host cells and also to the parasite load used.

In order to verify the obtained results the same experiments on two strains of inbred mice, characterized by low (BALB/c) or high (C57BL/6) innate susceptibility to *T. gondii* infection were performed. We analyzed the effect of phytoecdysteroids on the parasite proliferation in murine splenocytes derived from both uninfected and *T. gondii*-infected animals. Chronic toxoplasmosis was induced by a challenge with the DX strain and the presence of tissue cysts in brains of infected mice was confirmed microscopically just before the isolation of spleen cells. The parasite load ranged from 450 to 600 cysts per brain. We observed that the intensity of parasite proliferation in host cells collected from both mouse strains correlated with the increasing concentration of α-ecdysone; however, only in the case of the group of uninfected BALB/c mice the proliferation increase was statistically significant (38.86 %, *p* < 0.05; Table 3).

**Table 3:** Effect of the studied phytoecdysteroids on the intensity of RH *T. gondii* proliferation

Cmpd.	C [μg/mL]	Proliferation of parasites [%] in mouse splenocytes		
		BALB/c		C57BL/6
		*Toxo*-positive (*N* = 10)	*Toxo*-negative (*N* = 14)	*Toxo*-negative (*N* = 10)
α-ecdysone	2	89.29 ± 5.90 ↓	67.77 ± 5.05^*^ ↓	89.69 ± 2.54 ↓
	10	91.17 ± 2.89 ↓ 10 %	75.69 ± 2.34^*^ ↓ 39 %	99.75 ± 2.57 ↓ 13 %
	20	99.29 ± 4.14 ↓	106.63 ± 2.67^*^ ↓	102.58 ± 1.85 ↓
20-hydroxy ecdysone	2	88.65 ± 2.55	80.81 ± 3.35^*^	99.30 ± 2.55
	20	88.82 ± 3.11	109.28 ± 2.11^*^	103.73 ± 3.94
	100	90.02 ± 0.52	92.05 ± 2.34	97.76 ± 1.85
sulfadiazine	2	92.91 ± 5.11	78.34 ± 5.14^*^	85.79 ± 1.55^*^
	20	94.18 ± 2.35	70.87 ± 3.11^*^	74.65 ± 2.48^*^
	100	81.48 ± 1.52	65.32 ± 3.09^*^	62.31 ± 2.88^*^
ConA	2.5	192.60 ± 9.65^*^	151.48 ± 8.97^*^	191.62 ± 9.97^*^

Parasite proliferation was tested on splenocytes obtained from BALB/c mice previously infected with DX *T. gondii* (*Toxo*-positive), BALB/c and C57BL/6 uninfected (*Toxo*-negative) mice, using [^3^H] uracil incorporation. The statistically significant differences (*p* < 0.05) were labeled with an asterisk (*); ↓ indicates an increasing pool of protozoans with the increasing concentration of phytoecdysteroids in the splenocyte cultures

Ecdysteroids have been reported to exhibit a wide array of positive pharmacological effects on mammals, including carbohydrate, lipid and protein metabolism. Hence, multiple ecdysteroid-containing preparations are already commercially available, for instance as dietary supplements for sportsmen. Since ecdysteroids are considered as potential therapeutic drugs, it is essential to determine their effect on various pathogens. In general, the impact of phytoecdysteroids on parasites is poorly understood and does not explain the mechanisms of their action, which appears to vary in different parasites. While 20-hydroxyecdysone showed a significant inhibition of another apicomplexan parasite *Babesia gibsoni* [24], our results surprisingly demonstrated that phytoecdysteroids did not inhibit, but even promoted the multiplication of *Toxoplasma gondii* both in the human and murine immunocompetent cells. These observations are reflected by literature data referring to parasites such as *Trypanosoma cruzi*, which cause trypanosomiasis (Chagas disease) in humans and animals [37]. Barker et al. [38] reported that ecdysone can play a hormonal role in filarial worms similar to that found in insects. Namely, ecdysone was found to stimulate the microfilarial release in *Brugia pahangi* (a filarial worm of dogs and cats) and to control the meiotic reinitiation in the oocytes of *Dirofilaria immitis* (a roundworm spread by mosquitoes). It has been also demonstrated that *Onchocerca volvulus* and *Onchocerca lienalis* microfilariae increase their metabolic activity when treated *in vitro* with 20-hydroxyecdysone [39]. In the light of these observations, it is particularly important to consider the effect of ecdysteroids on immunocompromised patients, such as HIV-infected individuals, which are in a particular danger of developing a superinfection. Analogously to HIV-associated toxoplasmosis, patients infected with human T-cell leukaemia virus type I (HTLV-1) develop frequently a severe strongyloidiasis caused by a nematode *Strongyloides stercoralis* [40]. Although the moulting process in parasitic nematodes is very complex being possibly regulated by more than one transcriptional cascade, there is evidence that steroids, including ecdysteroids, may up-regulate the moulting signalling within the *S. stercoralis* larvae and it might be caused by a presence of a nuclear hormone receptor of the steroid/thyroid hormone-receptor superfamily [41, 42].

Ecdysteroids display hormonal function in insects (moulting hormones). However, their pharmacological effects on humans and animals cannot be simply considered as hormonal activity. The use of phytoecdysteroids in humans and animals has been proposed on the basis of their supposed interference with steroidal metabolic pathways of mammals. While the main focus remains on estrogenic and anabolic effects in humans [43] and domestic animals [44, 45], little has been published on these compounds’ immunomodulatory and anti-inflammatory potential. Trenin and Volodin [46] described 20-hydroxyecdysone as a human lymphocyte and neutrophil modulator. 20-hydroxyecdysone was also revealed to modulate the fluoride-stimulated respiratory burst of human neutrophils in the same manner as water soluble antioxidants. Moreover, 20-hydroxyecdysone dramatically attenuated renal injury in diabetes model. The authors concluded that 20-hydroxyecdysone might act through suppressing post-receptor signalling of TGF-β1. [47]. On the contrary, Harmatha et al. [48] found that 20-hydroxyecdysone did not influence the intensity of nitric oxide biosynthesis in mouse resident peritoneal macrophages stimulated by interferon-γ, and lipopolysaccharide. Similarly, Tanaguchi et al. [49] also did not observe any anti-inflammatory effect of 20-hydroxyecdysone in rats. Data obtained by Peschel et al. [50] in the HeLa-IL-6 model (cells stably transfected with an IL-6-bound reporter gen) implied a 20-hydroxyecdysone caused inhibition of nuclear factor - kappa B (NF-κB), which plays a central role in cellular immune response, inflammation and cell fate. The situation is different when standard human epithelial cells are infected with intracellular parasite such as *T. gondii*. Kim et al. [51] reported that *T. gondii* RH strain infected HeLa cells induced NF-κB and increased the expression of two chemokines: IL-8 and monocyte chemotactic protein-1 (MCP-1) mRNA. Moreover, the addition of IL-1α to *T. gondii* cultures increased the activation of NF-κB and IL-8 transcriptional reporters, compared to tachyzoite-infected cells without IL-1α treatment. However, the described phenomenon is not unequivocal because various studies showed that *Toxoplasma* has developed many strategies that can modulate the host NF-κB pathway. Evidence currently exists for both inhibition [52, 53] or activation [54–57] of the NF-κB pathway in host cell infected by *Toxoplasma* resulting in suppressed proinflammatory cytokine expression and enhanced survival of the pathogen or inhibition of apoptosis, an important defence mechanism against intracellular pathogens. The described results were dependent for example on whether *in vivo* or *in vitro* conditions were analyzed [53], what type of strain and techniques were used in the experiments. For example, infection with type I strain inhibited the NF-κB pathway and down-regulated the production of IL-12, thus limiting protective T helper 1(Th1)-type cytokine (IFN-γ, IL-2) response [52, 58] while *Toxoplasma* type II parasites activated the NF-κB p65 subunit [54]. It was confirmed that the observed differences were related to the various expression levels of ROP18 [52] or GRA15 [54] proteins, being some of the crucial antigens in *Toxoplasma* pathogenesis.

In the next step of our study the concentrations of crucial protective cytokines in *T. gondii* infection, IFN-γ, IL-2 and IL-10, were quantified in the post-culture supernatants of the human PBMCs (Tables 4 and 5) and mouse splenocytes (Tables 6 and 7) infected with *T. gondii* (Tables 5 and 7, respectively) or non-infected (Tables 4 and 6, respectively), and cultivated in the presence of phytoecdysteroids in the concentration range of 2–100 μg/ml (Tables 4, 5, 6 and 7). Treatment of human (Table 4) and mouse (Table 6) host cells with both tested ecdysteroids did not significantly change their secretory activity and the levels of IFN-γ and IL-2 as well as IL-10 were similar in cultures supplemented with ecdysteroids as compared to adequate controls without the compounds. The same results were observed in human PBMCs (Table 5) and mouse splenocytes (Table 7) infected *in vitro* by *T. gondii* RH tachyzoites. The host status: infected (BALB/c mice) vs. non-infected (BALB/c and C57BL/6 mice) (Tables 6 and 7) and phytoecdysteroids responder (humans, group A) vs. non-responder (humans, group B) (Table 4 and 5) did not appear to be important factors influencing cytokines secretion. But comparison of cytokine levels in control cultures of uninfected and not stimulated with phytoecdysteroids (Tables 4 and 6) vs*. Toxoplasma* infected and not stimulated with phytoecdysteroids host cells shows that *T. gondii* RH strain aroused IFN-γ and IL-10 production (Tables 5 and 7). This result is consistent with another observation that *in vitro* infection with type I strain did not inhibit NF-κB p65 but activated c-Rel nuclear translocation [54], an important host additional signaling pathway in the regulation of inflammatory, immune, and anti-apoptotic responses. The simultaneous presence of another stimulator such as phytoecdysteroids did not affect the cytokines release. According to our results presented in Tables 4, 5, 6 and 7, the tested phytoecdysteroids alone or in *Toxoplasma* co-cultures did not stimulate the *in vitro* secretion of the essential cytokines, either by human or by murine immune cells involved in an effective intracellular killing of the parasite. It is highly probable that, as in the case of HeLa [50], phytoecdysteroids inhibit NF-κB which is required for the production of cytokines such as IFN-γ or IL-12 [59]. The observed lack of *Toxoplasma* proliferation in human PBMCs in the presence of ConA (Table 2) correlated with the lack of IL-10 production but concomitantly with a statistically significant increase in IFN-γ level (Table 5). Interestingly, only in the phytoecdysteroids concentration of 20 μg/mL (Table 7), the levels of IFN-γ in the cultures of *T. gondii*-infected spleen cells derived from *T. gondii*-negative BALB/c mice were significantly lower (*p* < 0.05) than in controls and in other test cultures supplemented with ecdysteroids. Lower levels of IFN-γ were associated with higher parasite proliferation (Table 3).

**Table 4:** Cytokine profiles corresponding to human PBMCs cultured in the presence of phytoecdysteroids or concanavalin A

		Cytokine levels [pg/mL]							
PBMCs		control	α-ecdysone			20-hydroxyecdysone			ConA
		IFN-γ							
		0	2	10	20	2	20	100	2.5
A.	mean	126.75	132.52	129.92	125.68	127.04	124.58	131.26	2 661.02*
N = 52	SD	106.20	121.25	114.94	114.31	123.67	116.29	127.42	1197.77
	median	118.26	127.32	120.45	121.71	120.44	122.88	212.62	2 238.19
B.	mean	11.96	7.85	10.86	11.23	7.12	7.74	9.96	1003.35*
N = 8	SD	10.16	1.94	4.32	4.25	1.57	0.97	5.04	414.06
	median	7.21	7.97	9.32	9.63	7.07	7.75	7.51	107.77
		IL-10							
A.	mean	240.76	255.75	297.50	311.00	255.81	296.75	301.58	2758.74*
N = 48	SD	206.93	202.64	236.93	253.40	183.88	183.35	243.97	1075.34
	median	143.54	209.02	199.84	286.51	254.64	286.36	327.35	2849.68
B.	mean	13.12	8.56	8.66	7.23	9.89	8.93	11.30	606.68*
N = 9	SD	3.09	1.39	2.25	1.50	2.43	3.95	7.10	196.97
	median	13.00	8.79	8.79	7.60	10.57	7.90	8.79	574.96

PBMCs were derived from individuals who responded (A) or did not respond (B) to phytoecdysteroids. The statistically significant differences (*p* < 0.05) were labeled with an asterisk (*)

**Table 5:** Cytokine profiles corresponding to human PBMCs infected by *T. gondii* and incubated with phytoecdysteroids or concanavalin A

		Cytokine levels [pg/mL]							
PBMCs and *Toxoplasma*		control	α-ecdysone			20-hydroxyecdysone			ConA
		IFN-γ							
		0	2	10	20	2	20	100	2.5
A.	mean	579.94	668.42	644.86	601.85	612.61	603.78	615.51	1040.90*
N = 49	SD	552.02	541.49	515.38	500.54	540.02	524.31	522.29	543.24
	median	362.98	441.04	448.27	494.70	464.31	422.97	492.39	941.43
B.	mean	58.30	91.36	79.17	74.69	67.42	64.34	75.88	171.39*
N = 10	SD	35.80	65.93	50.10	46.09	35.78	40.41	50.66	68.67
	median	61.19	68.31	71.24	83.38	65.14	59.40	75.96	163.96
		IL-10							
A.	mean	622.90	542.24	616.43	706.24	608.92	660.99	623.21	728.45
N = 46	SD	309.04	338.16	292.83	350.71	311.57	309.01	328.14	589.63
	median	521.62	455.37	491.01	566.36	562.35	523.13	500.08	681.18
B.	mean	68.94	46.73	65.46	81.36	51.35	57.03	62.39	88.98
N = 12	SD	51.28	33.14	44.68	46.89	31.61	41.42	45.79	51.47
	median	41.48	38.49	40.54	56.04	40.38	37.97	39.59	68.94

PBMCs were derived from individuals who responded (A) or did not respond (B) to phytoecdysteroids. The statistically significant differences (*p* < 0.05) were labeled with an asterisk (*)

**Table 6:** Cytokine profiles corresponding to the mice splenocytes cultivated in the presence of phytoecdysteroids and concanavalin A

		Cytokine levels [pg/mL]							
Splenocytes		control	α-ecdysone			20-hydroxyecdysone			ConA
		IFN-γ							
		0	2	10	20	2	20	100	2.5
BALB/c	mean	752.33	606.89	552.24	667.61	716.58	709.40	663.52	1997.97*
N = 12	SD	634.53	423.51	438.02	599.92	452.11	568.89	409.02	369.25
negative	median	48.28	566.79	498.71	632.57	666.49	657.71	565.57	1857.23
BALB/c	mean	1735.99	1000.17	1168.71	1057.95	1540.23	1661.91	1560.54	4307.02*
N = 9	SD	714.81	687.62	843.80	901.84	572.05	604.58	633.65	82.08
positive	median	1848.28	878.07	976.23	819.85	1385.37	1485.31	1439.19	4292.66
		IL-10							
BALB/c	mean	19.49	20.84	18.87	19.60	21.39	19.20	20.54	1987.46*
N = 12	SD	9.08	13.12	7.80	10.45	8.12	8.85	9.55	340.02
negative	median	16.30	18.58	16.08	17.56	19.86	17.48	18.96	1883.19
BALB/c	mean	1344.07	1351.45	1385.59	1426.28	1550.71	1390.64	1437.98	2531.09*
N = 9	SD	583.19	543.89	550.90	522.69	506.47	494.77	521.20	661.58
positive	median	972.59	975.12	1006.89	1084.91	1228.71	1158.47	1079.78	2878.76
		IL-2							
BALB/c	mean	11.58	9.85	9.04	9.64	9.54	10.63	9.71	159.12*
N = 10	SD	9.76	2,55	3.32	3.21	3.82	3.79	3.23	30.02
negative	median	10.03	8.01	7.96	7.86	7.32	8.56	8.56	143.45
BALB/c	mean	16.22	21.82	15.88	19.91	19.31	19.80	20.98	276.97*
N = 10	SD	10.25	11.48	8.83	13.63	15.55	12.89	13.42	42.75
positive	median	15.25	17.77	14.25	16.45	13.55	15.50	19.05	290.12

Splenocytes were derived from BALB/c mice uninfected (*T. gondii*-seronegative) and chronically infected with *T. gondii* DX strain (*T. gondii*-seropositive). The statistically significant differences (*p* < 0.05) were labeled with an asterisk (*)

**Table 7:** Cytokine profiles corresponding to the splenocytes infected by *T. gondii* and cultivated in the presence of phytoecdysteroids or concanavalin A

		Cytokine levels [pg/mL]							
Splenocytes and *toxoplasma*		control	α-ecdysone			20-hydroxyecdysone			ConA
		IFN-γ							
		0	2	10	20	2	20	100	2.5
BALB/c	mean	1776.41	1748.22	1809.40	1263.67*	1431.23	908.90*	1860.88	2236.53*
N = 14	SD	672.75	452.11	568.89	109.02	760.64	200.90	1275.54	469.06
negative	median	1626.60	1666.49	1884.71	1265.57	1303.56	898.47	1854.40	1952.70
BALB/c	mean	2081.82	2983.11	2907.12	2912.51	2965.06	2884.66	2950.60	4159.40*
N = 10	SD	1253.55	1390.12	1445.01	1497.61	1541.11	1531.37	1477.52	322.44
positive	median	1604.36	3972.21	3812.13	3954.39	4070.46	3987.08	3984.11	4198.95
C57Bl/6	mean	816.88	790.06	749.12	827.65	639.40	652.87	765.19	3569.88*
N = 10	SD	326.21	278.98	237.76	232.67	232.76	245.14	219.75	378.49
negative	median	787.18	698.90	678.65	743.09	606.98	120.28	136.92	3339.78
		IL-10							
BALB/c	mean	534.73	531.39	538.11	639.41	631.99	535.42	631.71	1987.46*
N = 14	SD	369.76	367.85	317.15	411.98	310.57	410.32	418.65	440.02
negative	median	532.30	531.08	532.00	636.93	629.86	533.53	528.94	2083.19
BALB/c	mean	1836.67	2054.01	1783.78	1907.01	2252.42	1981.89	1942.38	3157.88*
N = 10	SD	387.73	579.95	716.93	499.15	592.62	392.76	433.08	1186.99
positive	median	1638.48	1952.01	1540.04	1691.38	1946.47	1760.87	1821.10	3615.60
C57Bl/6	mean	28.73	30.68	33.36	28.94	29.59	31.75	25.98	516.83*
N = 9	SD	20.33	24.72	27.19	19.04	17.88	16.70	19.85	378.49
negative	median	25.46	28.56	29.46	25.98	26.58	29.87	34.56	501.78
		IL-2							
BALB/c	mean	22.98	20.22	20.69	23.24	21.45	26.25	24.47	163.68*
N = 10	SD	20.69	19.85	19.87	18.23	16.59	20.69	20.58	54,76
negative	median	21.20	20.30	19.58	19.23	19.96	19.65	22.87	139.78
BALB/c	mean	124.42	151.08	130.26	140.97	149.61	146.26	166.44	339.63*
N = 10	SD	69.58	64.59	56.79	56.89	76.40	72.27	69.21	14.84
positive	median	87.18	120.20	116.25	125.37	131.56	120.28	136.92	339.78
C57Bl/6	mean	12.54	10.02	10.17	10.29	11.42	12.26	11.87	163.68*
N = 10	SD	10.70	9.08	9.57	8.92	6.38	10.84	10.10	54,76
negative	median	10.25	10.00	9.25	9.98	10.25	10.24	10.36	139.78

Splenocytes were derived from uninfected (*T. gondii*-seronegative) BALB/c or C57BL/6 as well as from chronically infected with *T. gondii* DX strain (*T. gondii*-seropositive) BALB/c mice. The statistically significant differences (*p* < 0.05) were labeled with an asterisk (*)

## Conclusions

**Fig. 3:**
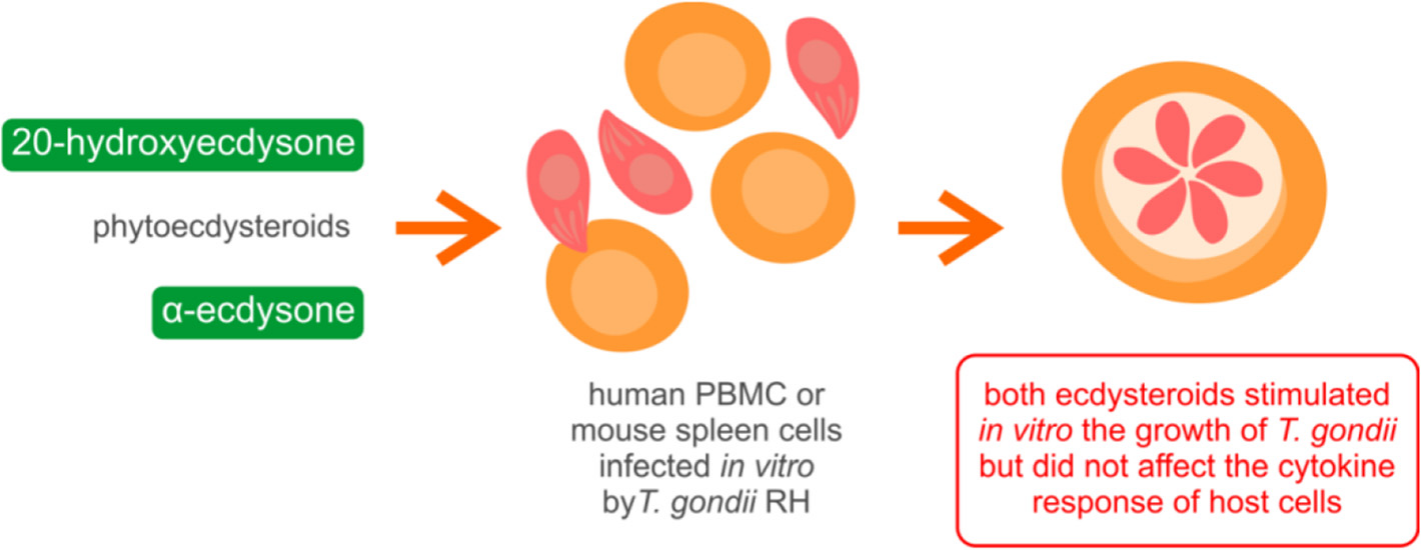
Graphical Conclusions

Judging by the effect of phytoecdysteroids on the *T. gondii* proliferation, demonstrated for the first time in this study, it seems that these compounds should not be taken into consideration as potential medications to treat toxoplasmosis. Phytoecdysteroids included in the food are most likely not harmful for human or animal health but certain nutrients containing ecdysteroids at high concentrations could promote *T. gondii* proliferation in chronically infected and immunocompromised individuals. In order to assess the real impact of ecdysteroids on the course of natural *T. gondii* invasion, *in vivo* research should be undertaken because it cannot be ruled out that the *in vivo* effect will be different than the *in vitro* one. However, taking into account the possible stimulating effect of ecdysteroids on some opportunistic parasites (such as *Toxoplasma* or *Strongyloides*) further studies are necessary and should focus on the mechanisms of their action, which directly or indirectly enhance the parasite growth. Since ecdysteroids are considered as potential drugs, it is essential to determine their effect on various parasitic pathogens, which may infect the host at the same time, especially in immunocompromised individuals (Fig. 3).
